# Regulator of Ribosome Synthesis 1 (RRS1) Stabilizes GRP78 and Promotes Breast Cancer Progression

**DOI:** 10.3390/molecules29051051

**Published:** 2024-02-28

**Authors:** Wenjing Sun, Junying Song, Qinglan Wu, Lin Deng, Tenglong Zhang, Li Zhang, Yanan Hua, Yi Cao, Lin Hou

**Affiliations:** 1 Department of Biochemistry and Molecular Biology, School of Basic Medicine, Qingdao University, Qingdao 266011, China; 2021020878@qdu.edu.cn (W.S.); 2020010052@qdu.edu.cn (J.S.); 2020020815@qdu.edu.cn (Q.W.); 2021020877@qdu.edu.cn (T.Z.); 2 Wanzhou District Center for Disease Control, Chongqing 404100, China; 2020020820@qdu.edu.cn; 3 Experimental Center for Undergraduates of Pharmacy, School of Pharmacy, Qingdao University, Qingdao 266011, China; zhangli617@126.com; 4 Chongqing Key Laboratory of Sichuan-Chongqing Co-Construction for Diagnosis and Treatment of Infectious Diseases Integrated Traditional Chinese and Western Medicine, College of Medical Technology, Chengdu University of Traditional Chinese Medicine, Chengdu 611137, China; huayanan@cdutcm.edu.cn

**Keywords:** RRS1, GRP78, breast cancer, interaction

## Abstract

Regulator of ribosome synthesis 1 (RRS1), a crucial regulatory factor in ribosome biogenesis, exerts a remarkable impact on the progression of breast cancer (BC). However, the exact mechanisms and pathways have not yet been fully elucidated. To investigate the impact of RRS1 on BC growth and metastasis, along with its underlying mechanisms. We discovered that RRS1 is overexpressed in BC tissues and cell lines. This study aims to regulate the level of RRS1 through lentiviral transfection technology to explore its potential function in BC cells. Knockdown of RRS1 resulted in the inhibition of cell proliferation, invasion, and migration, whereas overexpression had the opposite effects. We firstly identified the interaction between RRS1 and Glucose-Regulated Protein 78 (GRP78) using Co-immunoprecipitation (Co-IP) combined with mass spectrometry analysis, providing evidences of co-localization and positive regulation between RRS1 and GRP78. We observed that RRS1 inhibited the degradation of GRP78 through the ubiquitin–proteasome pathway, resulting in the stabilization of GRP78. In addition, our findings suggested that RRS1 promoted BC progression by activating the GRP78-mediated phosphoinositide 3-kinase (PI3K)/protein kinase B (AKT) signaling pathway. In conclusion, this newly discovered RRS1/GRP78 signaling axis provides a molecular and theoretical basis for further exploring the mechanisms of breast cancer invasion and metastasis.

## 1. Introduction

Worldwide, breast cancer (BC) has become one of the most frequent cancers affecting women, posing a significant threat to female health, and standing as a leading cause of death among women [1]. As age advances, both the incidence and mortality rates of BC are increasing. Reports indicate a gradual rise in the number of new cases and deaths related to BC in women under the age of 45 [2,3,4]. Metastasis is responsible for the majority of BC-related deaths, accounting for over 90% of the total. This type of tumor primarily metastasizes to vital organs, with the liver, lungs, bones, and brain being the most common sites [5,6]. Currently, surgery and radiotherapy have been the primary clinical interventions for BC, complemented by chemotherapy, endocrine therapy, and immunotherapy. However, drug resistance together with the highly metastatic nature of BC limit the effectiveness of these treatment modalities, leading to relatively low survival rates among patients [7]. Hence, it is crucial to investigate the mechanisms of BC and search for novel targets for its treatment.

Regulator of ribosome synthesis 1 (RRS1), initially identified in yeast, plays a crucial role as a regulatory factor in ribosome synthesis [8]. It is a multifunctional protein predominantly localized in the nucleolus and endoplasmic reticulum (ER) [9]. Functioning as a regulatory protein for ribosome synthesis, RRS1 is involved in pre-rRNA processing and the transportation of 60S ribosomal subunits between the nucleolus and the cytoplasm [10]. Apart from its involvement in ribosome biogenesis, RRS1 is also associated with chromosomal rearrangement [11], telomere aggregation [12], and cellular senescence [13]. The association between RRS1 and diseases was first established in Huntington’s disease, wherein RRS1 significantly contributes to pathogenesis by promoting ER stress [14]. In the past few years, the role of the RRS1 gene in tumors has garnered attention. For instance, Wu et al. demonstrated that knockdown of the RRS1 gene inhibited the proliferation rates as well as the tumorigenesis of colorectal cancer cells by blocking G2/M phase progression and angiogenesis [15]. Similarly, Yan et al. observed that suppressing the RRS1 gene in retinoblastoma cells inhibited cell proliferation and invasion through the protein kinase B (AKT)/mechanistic target of the rapamycin (mTOR) pathway [16]. Our research team has previously demonstrated that RRS1 expression was linked to a poor prognosis in BC and might promote the proliferation of BC cells via ribosomal protein L11 (RPL11)/murine double minute 2 (MDM2)-mediated p53 activation [10]. Therefore, it can be speculated that the occurrence and progression of malignancies are strongly correlated with RRS1. While our previous work focused on the impact of RRS1 on BC cell proliferation, research on the relationship between the RRS1 gene and BC remains limited. Therefore, our investigation was aimed to analyze the impact of RRS1 on BC invasion and metastasis, along with its underlying mechanisms, providing potential targets and novel insights for the prevention and treatment of BC in future.

Glucose-regulated protein 78 (GRP78), alternatively referred to as heat shock protein family A (Hsp70) member 5 (HSPA5) or binding immunoglobulin protein (BIP), is a heat shock protein family member that is necessary for the endoplasmic reticulum (ER) to function in response to stress [17]. As a key regulator of cellular homeostasis, GRP78 is primarily localized in the cytoplasm and ER, where it actively participates in protein folding and the degradation of misfolded proteins [18]. An extensive body of research has consistently demonstrated the critical involvement of GRP78 in multiple essential functions related to cancer, including tumor cell proliferation, differentiation, migration, apoptosis, and drug resistance [19,20,21,22]. The aberrant upregulation of GRP78 expression is closely associated with tumor resistance, an elevated risk of cancer recurrence, and diminished overall survival across diverse types of cancer [23,24,25,26,27]. Hence, GRP78 is considered a promising target for cancer therapy.

Our investigation found that RRS1 knockdown effectively suppressed the proliferation, invasion, and the migration of BC cells. To unravel the underlying mechanism for RRS1 among BC cells, we employed Co-immunoprecipitation (Co-IP) combined with mass spectrometry (MS) analysis and, for the first time, revealed the interaction between RRS1 and GRP78, which significantly enhanced the stability of the GRP78 protein. Additionally, RRS1 could activate the phosphoinositide 3-kinase (PI3K)/AKT pathway through its interaction with GRP78, thereby promoting the progression of BC. Therefore, the principal objective of this study was to comprehensively analyze the function and the mechanism of RRS1 in BC metastasis, providing scientific foundation for considering RRS1 as a highly promising target for clinical interventions and the diagnosis of BC.

## 2. Results

### 2.1. High Expression of RRS1 in Human BC Tissues and Cell Lines

**Figure 1:**
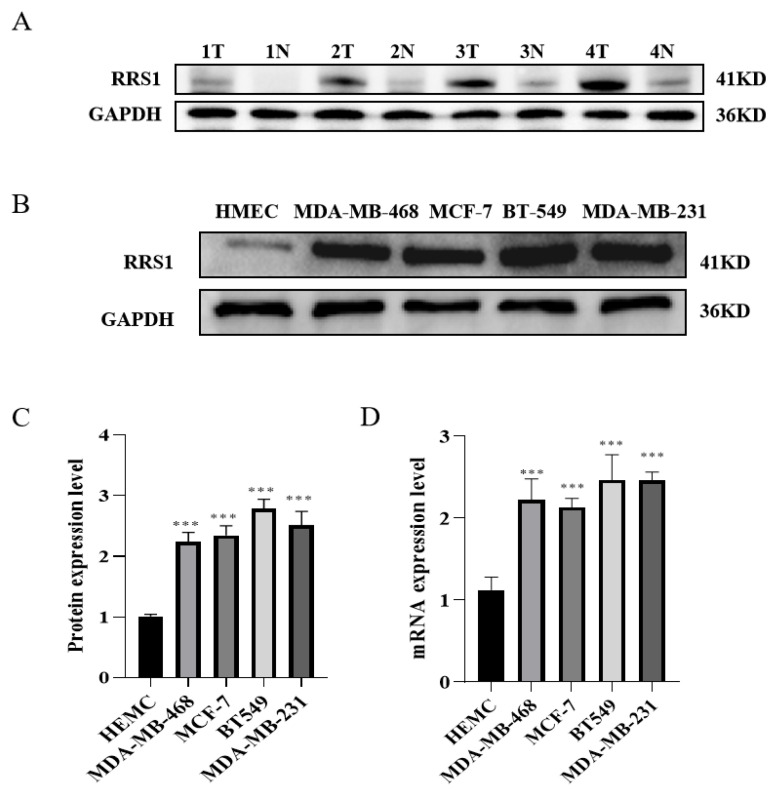
High expression of RRS1 in human BC tissues and cell lines. (**A**) RRS1 protein expression in tissues from breast cancer, T: tumor, N: non-tumor. (**B**,**C**) RRS1 protein expression in normal breast epithelial cells and BC cell lines. (**D**) RRS1 mRNA expression in normal breast epithelial cells and BC cell lines. The data are presented as the means ± standard deviations (SD) from at least three independent experiments. ***, *p* < 0.001.

To investigate the expression of RRS1 in BC tissues, we assessed its levels using WB in both BC tissues and nearby tissues. The results showed a significantly higher protein expression level of RRS1 in BC tissues in contrast with their corresponding adjacent tissues (Figure 1A). To deeply investigate RRS1 expression among BC cells, WB together with RT-qPCR was adopted for the detection of the mRNA and protein expressions of RRS1 among human microvascular endothelial cells (HMECs) as well as four different types of BC cells (MDA-MB-231, BT-549, MDA-MB-468, and MCF-7). These findings indicated that the four BC cell lines obviously increased the mRNA and protein expression levels of RRS1 compared to the HMEC cell line (Figure 1B–D).

### 2.2. RRS1 Promoted the Proliferation, Migration, and Invasion in BT-549 and MDA-MB-231 Cells

**Figure 2:**
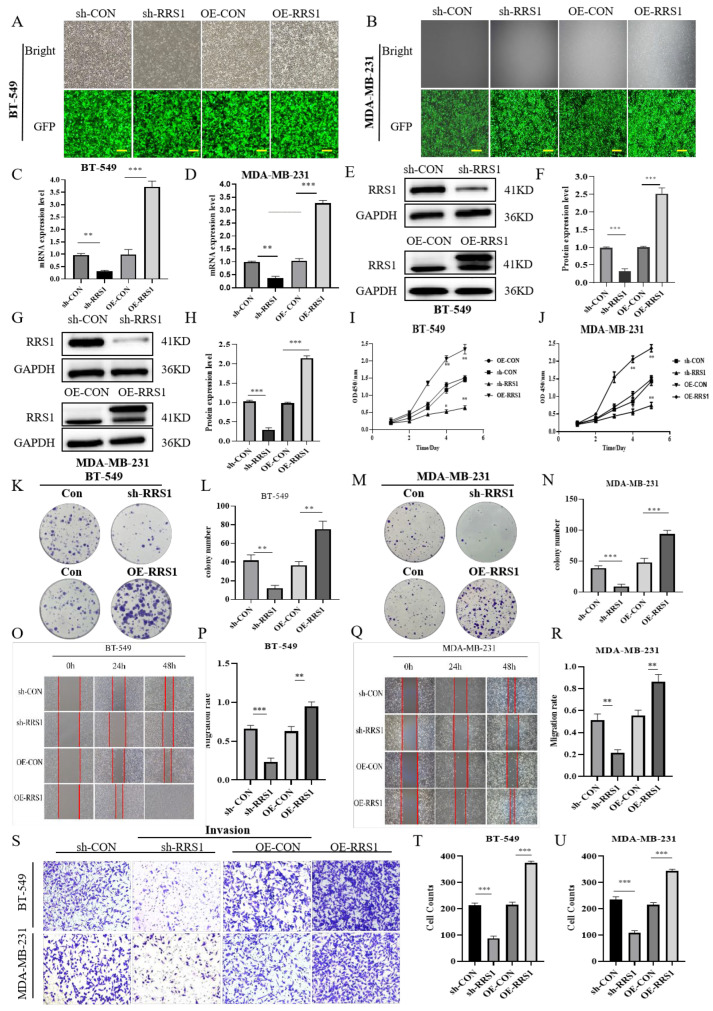
RRS1 promoted the proliferation, migration, and invasion in BT-549 and MDA-MB-231 cells. (**A**,**B**) Fluorescence intensity of lentivirus-infected BT-549 cells and MDA-MB-231cells was observed using a fluorescence microscope. (**C**,**D**) Quantification of RRS1 mRNA expression levels in BT-549 and MDA-MB-231 cells using RT-qPCR. (**E**–**H**) WB analysis of RRS1 protein expression in BT-549 and MDA-MB-231 cells. (**I**,**J**) CCK8 assay to evaluate the growth of BT-549 and MDA-MB-231 cells. (**K**–**N**) Colony formation assay to investigate the impacts of RRS1 on cell growth. (**O**–**R**) Scratch assay to determine the impact of RRS1 knockdown or overexpression on the migration ability of BT-549 and MDA-MB-231 cells. (**S**–**U**) Transwell assay to analyze the invasive potential of BT-549 and MDA-MB-231 cells. The data are presented as the means ± standard deviations (SD) from at least three independent experiments. *, *p* < 0.05; **, *p* < 0.01; ***, *p* < 0.001.

The aforementioned experimental results demonstrated RRS1 expression is increased in BC cell lines, which were critical for BC development. To deeply explore the role of RRS1 on the modulation of BC cells, two BC cell lines (BT-549 cells together with MDA-MB-231 cells) with high RRS1 expression were selected for related experiments in this study. BT-549 and MDA-MB-231 cells were infected with shRNA targeting RRS1 (sh-RRS1), control shRNA (sh-CON), scrambled control (OE-CON), and overexpressed RRS1 (OE-RRS1) through lentiviral infection technology, respectively. After 72 h of infection, inverted fluorescence microscopy was used to observe and record fluorescence intensity, preliminary assessing the infection efficiency (Figure 2A,B) and the infection efficiency reached more than 80%, and subsequent experiments were conducted. Subsequently, WB and RT-qPCR analyses were conducted on BT-549 and MDA-MB-231 cells to confirm successful transfection by examining protein and mRNA expression levels of RRS1. The knockdown or overexpression efficiency was validated by the reduced or increased protein and mRNA levels of RRS1. Results of the study demonstrated a significant increase in RRS1 protein and mRNA expression in the overexpressed cell lines, while showing a substantial downregulation in the knockdown cell lines (Figure 2C–H). The influence of RRS1 on the proliferation ability of BT-549 and MDA-MB-231 cells was evaluated using cell proliferation and CFAs. Knockdown of RRS1 significantly inhibited cell proliferation and weakened the colony-forming capacity of BT-549 and MDA-MB-231 cells, whereas overexpression of RRS1 produced opposite effects (Figure 2I–N). Transwell invasion assays along with scratch assays were carried out to evaluate RRS1’s influence on cancer cell invasion and migration. Knocking down RRS1 dramatically decreased cell invasion and migration, whereas RRS1 overexpression had the opposite effects (Figure 2O–U).

### 2.3. RRS1 Interacted with GRP78 and Regulated Its Protein Levels

**Figure 3:**
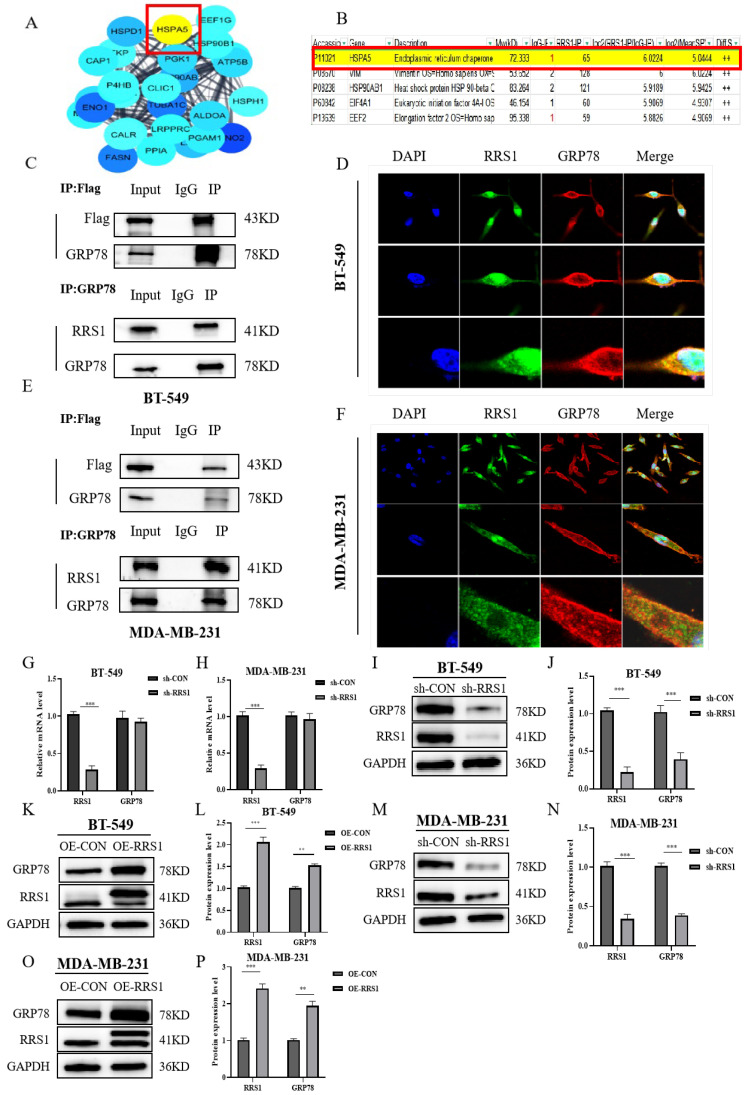
RRS1 interacted with GRP78 and regulated its protein levels. (**A**) Identification of potential proteins interacting with RRS1. (**B**) Co-immunoprecipitation assay combined with mass spectrometry (MS) analysis. (**C**) Interaction between RRS1 and GRP78 in BT-549 cells. (**D**) IF analysis showing co-localization of GRP78 (red fluorescence), RRS1 (green fluorescence), and DAPI (blue fluorescence) in BT-549 cells. (**E**) Interaction between RRS1 and GRP78 in MDA-MB-231 cells. (**F**) IF analysis showing co-localization of GRP78 (red fluorescence), RRS1 (green fluorescence), and DAPI (blue fluorescence) in MDA-MB-231 cells. (**G**,**H**) mRNA levels of RRS1 and GRP78 after RRS1 knockdown. (**I**,**J**) WB analysis of GRP78 protein expression in BT-549 cells following RRS1 knockdown. (**K**,**L**) WB analysis of GRP78 protein expression in BT-549 cells following RRS1 overexpression. (**M**,**N**) WB analysis of GRP78 protein expression in the MDA-MB-231 cells following RRS1 knockdown. (**O,P**) WB analysis of GRP78 protein expression in the MDA-MB-231 cells following RRS1 overexpression. The data are presented as the means ± standard deviations (SD) from at least three independent experiments. **, *p* < 0.01; ***, *p* < 0.001.

To further investigate the molecular mechanisms by which RRS1 mediates BC progression, we initially identified proteins interacting with RRS1 through network databases (Figure 3A). Additionally, protein MS analysis revealed the presence of GRP78 known as HSPA5 (Figure 3B). This finding was confirmed by Co-IP experiments using anti-GRP78 antibodies, which successfully precipitated RRS1. Due to the limitations of RRS1 antibodies in Co-IP experiments, we utilized lentiviral infection with Flag-tagged overexpressed RRS1 in BC cells and performed IP using anti-Flag antibodies. The results demonstrated that Flag could precipitate GRP78, confirming the interaction between RRS1 and GRP78. Immunoprecipitation co-localization further supported this conclusion (Figure 3C–F). Breast cancer patients expressing high levels of GRP78 were more likely to develop metastasis and have a poor prognosis. GRP78 was highly expressed in BC cells [28]. Therefore, we speculated whether GRP78 was involved in the process of the proliferation, the invasion, alongside the migration of BC cells mediated by RRS1. To investigate any possible regulatory connection between RRS1 and GRP78, we initially knocked down RRS1 in BT-549 alongside MDA-MB-231 cells through lentiviral transfection and assessed GRP78 mRNA levels using RT-qPCR. The results showed that silencing RRS1 did not lead to any evident changes in GRP78 (Figure 3G–H). WB analysis of GRP78 protein levels revealed a significant decrease after RRS1 knockdown and an increase after RRS1 overexpression (Figure 3I–P). These data suggested that RRS1 interacted with GRP78 and positively regulated GRP78 protein expression.

### 2.4. RRS1 Maintained GRP78 Stability and Blocked Ubiquitination and Proteasomal Degradation of GRP78

**Figure 4:**
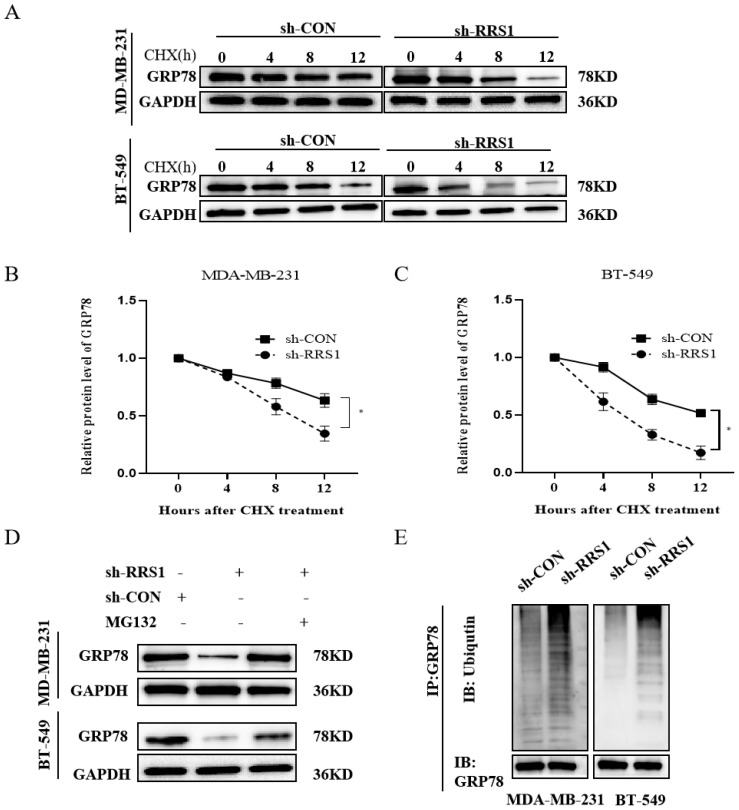
RRS1 maintained GRP78 stability and blocked ubiquitination and proteasomal degradation of GRP78. (**A**–**C**) Assessment of GRP78 stability in RRS1-knockdown BT-549 and MDA-MB-231 cells using WB analysis after treatment with 710 µM CHX for a specific duration. (**D**) Analysis of GRP78 protein levels in MG132-treated cells (10 µM for 6 h). (**E**) Immunoprecipitation analysis using anti-GRP78 antibodies followed by WB analysis with anti-ubiquitin antibodies. The data are presented as the means ± standard deviations (SD) from at least three independent experiments. *, *p* < 0.05.

To gain further insights into the relationship of RRS1 with GRP78 protein levels, we employed CHX, a known inhibitor of protein synthesis, to investigate whether RRS1 influences the content of GRP78 by modulating its stability. Our results showed that the knockdown of RRS1 caused a downregulation of GRP78 expression and a shortened half-life of the protein (Figure 4A–C). Additionally, we observed that the depletion of GRP78 protein induced by RRS1 knockdown could be reversed upon treatment with the proteasome inhibitor MG132 (Figure 4D). To thoroughly examine the possible influence of RRS1 on regulating the ubiquitination of GRP78 protein, MG132, a potent proteasome inhibitor, was added to both sh-CON and sh-RRS1 cells. Subsequently, anti-GRP78 antibodies were used to immunoprecipitate cell lysates, which were then subject to WB analysis using anti-ubiquitin antibodies. A substantial increase in GRP78 ubiquitination was observed when comparing sh-RRS1 cells to sh-CON cells (Figure 4F). Based on these findings, it could be inferred that RRS1 may be crucial for maintaining the stability of GRP78 by interfering with the ubiquitin–proteasome pathway, thereby preventing the degradation of GRP78.

### 2.5. RRS1 was Involved in the PI3K/AKT Signaling Pathway

**Figure 5:**
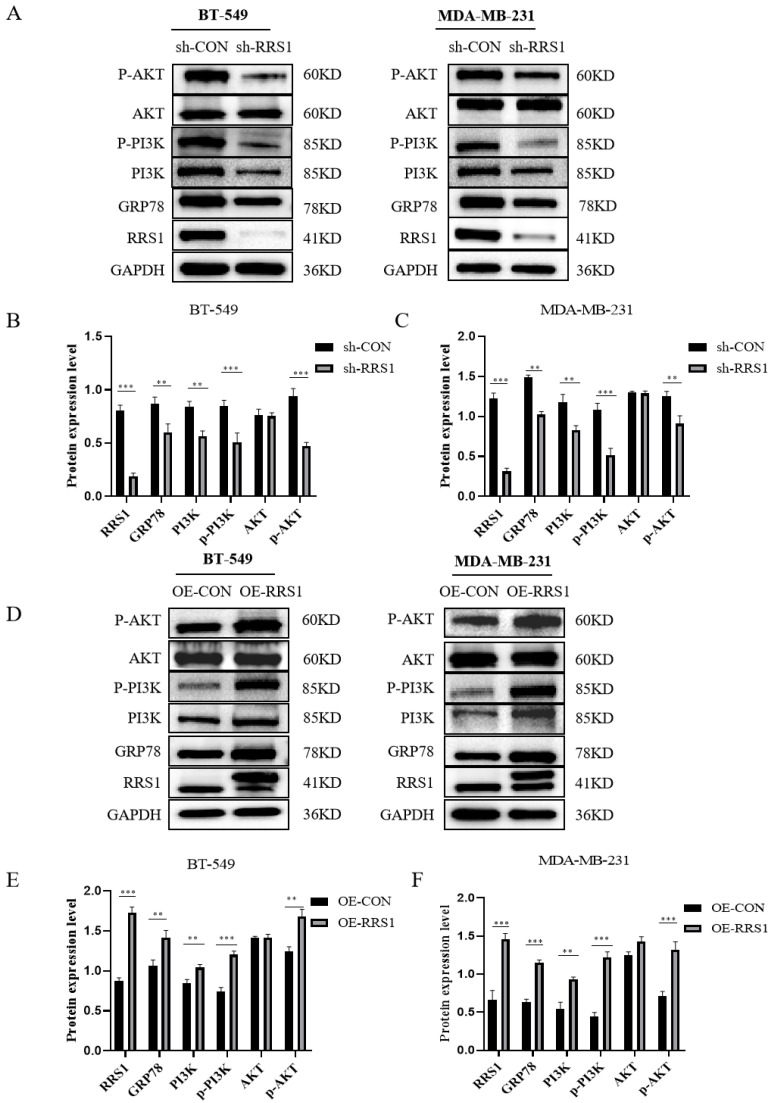
RRS1 was involved in the PI3K/AKT signaling pathway. (**A**–**C**) WB analysis of GRP78, PI3K, p-PI3K, AKT, and p-AKT expression among BT-549 and MDA-MB-231 cells after RRS1 knockdown. (**D**–**F**) WB analysis of GRP78, PI3K, p-PI3K, AKT, and p-AKT expression among BT-549 and MDA-MB-231 cells following RRS1 overexpression. The data are presented as the means ± standard deviations (SD) from at least three independent experiments. **, *p* < 0.01; ***, *p* < 0.001.

Proliferation and metastasis of BC could be significantly influenced by the PI3K/AKT pathway. Previous studies have demonstrated that GRP78 could promote the metastasis of lung cancer by triggering the PI3K/AKT pathway [29]. It has also been proved that GRP78 could bind to the p85 subunit, promoting the formation of phosphatidylinositol 3,4,5-trisphosphate (PIP3) and subsequently activating the AKT protein [30]. Due to the connection between RRS1 and the PI3K/AKT signaling pathway [31], we hypothesized that RRS1 might participate in this pathway through GRP78, thereby contributing to the promotion of BC progression. We assessed the expression of proteins linked to the PI3K/AKT pathway through WB analysis. Remarkably, inhibition of RRS1 decreased the levels of GRP78, PI3K, p-PI3K, as well as p-AKT (Figure 5A–C). Conversely, overexpression of RRS1 exhibited the opposite effects (Figure 5D–F). Collectively, these findings showed that the PI3K/AKT pathway contributed to the RRS1-induced pathological progression of BC.

### 2.6. RRS1 Regulated the GRP78-Mediated PI3K/AKT Signaling Pathway

**Figure 6:**
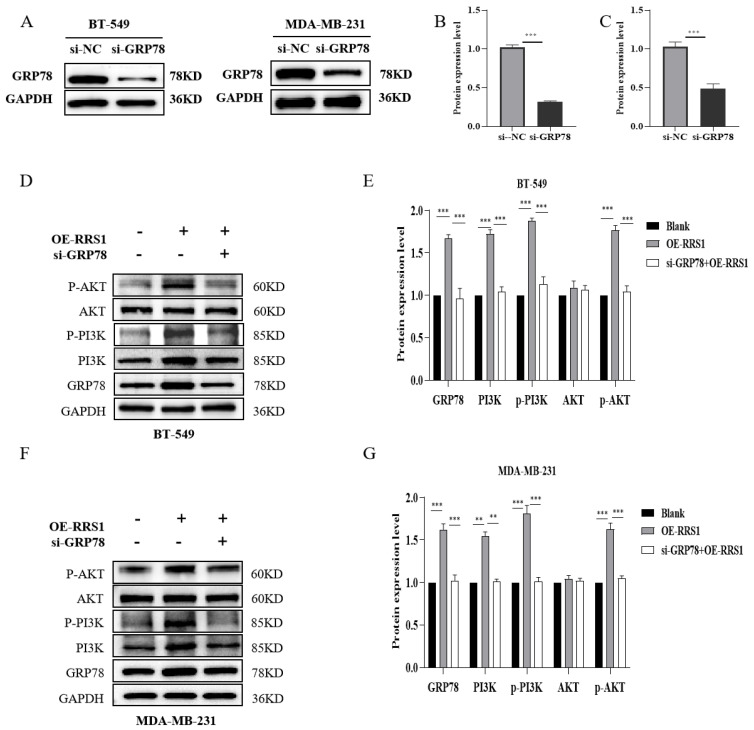
RRS1 regulated the GRP78-mediated PI3K/AKT signaling pathway. (**A**–**C**) WB analysis to determine the effectiveness of si-GRP78 interference. (**D**,**E**) WB analysis of GRP78, PI3K, p-PI3K, AKT, and p-AKT expression in BT-549 and OE-RRS1 cells treated with si-GRP78. (**F**,**G**) WB analysis of GRP78, PI3K, p-PI3K, AKT, and p-AKT expression in MDA-MB-231 and OE-RRS1 cells treated withsi-GRP78. The data are presented as the means ± standard deviations (SD) from at least three independent experiments. **, *p* < 0.01; ***, *p* < 0.001.

To further validate that RRS1 regulated the PI3K/AKT signaling pathway through GRP78, we downregulated GRP78 expression levels in BT-549 and MDA-MB-231 cells using siRNA interference. WB analysis confirmed the successful knockdown of GRP78 (Figure 6A–C). Subsequently, following lentiviral overexpression of RRS1, siRNA interference was utilized to suppress GRP78 expression. These results demonstrated that the increased levels of PI3K, p-PI3K, alongside p-AKT induced by RRS1 overexpression were reversed by the knockdown of GRP78 (Figure 6D–G). In summary, RRS1 regulated the PI3K/AKT pathway through GRP78, thereby promoting the progression of BC.

## 3. Discussion

BC has become one of the most frequent malignancies in adult females. Currently, the incidence of BC in young individuals has been increasing in China, resulting in a continuous rise in newly diagnosed cases [32]. Despite significant advancements in early diagnosis and standardized treatment, postoperative recurrence and metastasis remain major challenges that affect patient survival [33]. The complex process of BC metastasis and the unclear underlying mechanisms necessitate a more comprehensive understanding of the potential mechanisms underlying its occurrence and development.

Studies have indicated that RRS1 is a regulatory factor in ribosome biogenesis, playing a crucial role in the synthesis of ribosomes [8]. RRS1 recruits the 5S ribonucleoprotein (RNP) and the ribosome-forming factor 2 to facilitate the assembly of pre-60S ribosomal subunits, thereby promoting the maturation of 25S rRNA and regulating ribosome biosynthesis [10]. Increased ribosome biogenesis is necessary to maintain the elevated proliferation rate of tumor cells [34]. Recent research has implicated RRS1 in the development of several malignancies and it is significantly upregulated in various tumors [35,36,37,38]. The results of our previous studies have demonstrated the function of RRS1 in the growth of BC cells. However, limited research has been conducted on its role in BC cell invasion and metastasis. Our study explored RRS1 gene’s impact on the invasion and metastasis of BC, as well as its potential molecular mechanisms.

According to our research, RRS1 expression was obviously increased among BC tissues. Compared to normal breast epithelial cells, BC cell lines showed significantly higher levels of RRS1 expression. The Luminal A subtype exhibits high expression of estrogen receptor (ER) and progesterone receptor (PR), while lacking HER-2 expression. Additionally, it is characterized by a low proliferation rate and favorable prognosis [39]. Triple-negative breast cancer (TNBC) is characterized by the loss of HER-2, PR, and ER expression, and has strong chemotherapy resistance, metastasis, and invasiveness. Patients with triple-negative breast cancer have a poor prognosis and a high recurrence rate [40,41]. In this study, we selected bt-549 and MDA-MB-231 cell lines with high RRS1 expression for subsequent experiments. RRS1 promotes the migration, invasion, and proliferation of BC cells. This suggests that RRS1 plays an indispensable role in the occurrence and development of breast cancer.

Extensive research has demonstrated that GRP78 plays a significant cytoprotective role by inhibiting apoptosis and promoting metastasis in tumor cells [42,43]. Additionally, GRP78 is essential for the progression of various cancer types. For instance, the downregulated GRP78 has been observed to inhibit invasion in hepatocellular carcinoma [44], while abnormal expression of GRP78 affects the growth and survival of melanoma cells [45]. Evidence has suggested a worse prognosis in patients with BC who have increased levels of GRP78 [46,47], highlighting it as an underlying target for cancer therapy. In the present study, we discovered for the first time that RRS1 can interact with GRP78 referred to as HSPA5. However, our research revealed that RRS1 knockdown had no effect on GRP78 mRNA levels. RRS1 increased the levels of GRP78 protein in BC cells without altering its transcriptional activity. The research results showed that GRP78 can be polyubiquitinated and subsequently degraded by the ubiquitin proteasome system, thereby inhibiting cell migration and invasion [48,49,50]. Our findings revealed that the knockdown of RRS1 significantly reduced the half-life of GRP78 among BC cells. Notably, this study elucidated that RRS1 maintained the stability of GRP78 by interfering with the ubiquitin–proteasome pathway, thereby preventing GRP78 degradation.

The PI3K/AKT signaling pathway is crucial for distinct biological processes, such as glucose metabolism, apoptosis, cell proliferation, and cell migration [51,52,53]. Previous research have proved the relationship of RRS1 and GRP78 with this pathway [31,54]. Our study assessed the impact of RRS1 on the PI3K-AKT pathway. Consistently, our findings consistently indicated that the PI3K/AKT pathway was upregulated during RRS1-induced BC progression. To further validate whether RRS1 activates the PI3K/AKT pathway through GRP78, we utilized lentiviral infection technology to overexpress RRS1 and employed siRNA to downregulate GRP78 expression. Experimental evidence demonstrated that the knockdown of GRP78 could abolish the elevated protein level of either p-PI3K or p-AKT induced by RRS1 overexpression. Therefore, we concluded that RRS1 potentially modulated the PI3K/AKT pathway by GRP78.

However, there were still some limitations in our current experiments, and the precise regulatory mechanism underlying the interaction between RRS1 and GRP78 remains incompletely understood. Therefore, in subsequent investigations, we will further explore and augment our understanding of this molecular mechanism.

**Figure 7:**
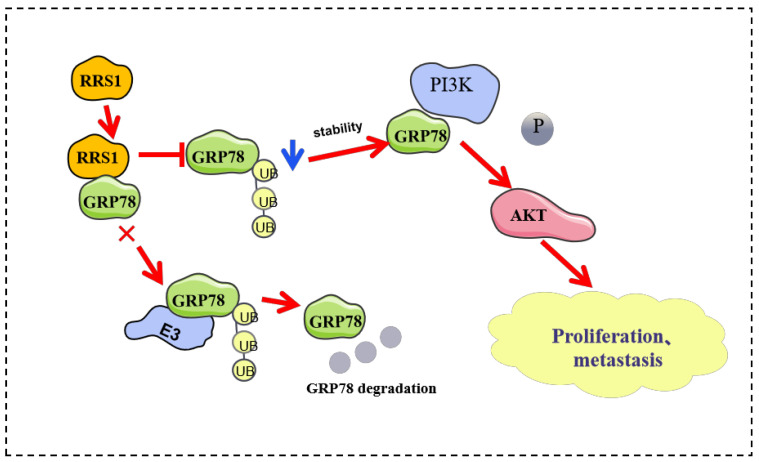
RRS1 activated the PI3K/AKT signaling pathway to promote the progression of BC through the stabilization of GRP78.

In this study, we identified a significant role of RRS1 in mediating the proliferation, invasion, as well as migration of BC cells. Furthermore, we have uncovered, for the first time, an interaction between RRS1 and GRP78. Specifically, RRS1 may increase the protein levels of GRP78 by inhibiting ubiquitination and proteasomal degradation. Additionally, RRS1 facilitated the progression of BC cells through modulating the activation of the PI3K/AKT pathway through GRP78 (Figure 7). Consequently, the identification of inhibitors that target RRS1 or disrupt the RRS1-GRP78 interaction may present promising avenues for the development of novel strategies in BC treatment.

## 4. Materials and Methods

### 4.1. Tissue Samples

Human tissue samples were obtained from the Department of Breast Surgery, Affiliated Hospital of Qingdao University. Specimens of primary BC tissue, as well as nearby tissues, were collected. After tumor tissues were isolated, they were immediately preserved in a specimen storage solution, labeled, and stored in a refrigerator under ultra-low-temperature conditions of −80 °C. The informed consent was obtained from each patient. This study was approved by the ethics committee (NO.QDU-HEC-2023066) prior to its initiation.

### 4.2. Cell Culture and Chemical Reagents

The MDA-MB-231, MDA-MB-468, BT-549, and MCF-7 BC cell lines were obtained from Wuhan Prolife Bioscience Technology Co., Ltd. (Wuhan, China). Among them, MDA-MB-231, BT-549, and MDA-MB-468 cells are classified as triple-negative breast cancer (TNBC) cells, characterized by the absence of hormone receptors such as progesterone receptor (PR), estrogen receptor (ER), and human epidermal growth factor receptor-2 (HER-2). On the other hand, MCF-7 is an in situ breast cancer cell line characterized by luminal epithelium A expressing positive progesterone receptor (PR(+)), estrogen receptor (ER(+)), and negative human epidermal growth factor receptor 2 (HER-2(-)). Normal breast epithelial cells (HEMC) were acquired from the Shanghai Biocell Bank of the Chinese Academy of Sciences. In addition, all cell lines were subjected to short tandem repeat (STR) analysis for identification. Routine quality control confirmed their negative status for mycoplasma contamination. Dulbecco’s modified Eagle medium (DMEM) with a high glucose concentration (SH30243.01), procured from Hyclone, was supplemented with a 10% heat-inactivated fetal bovine serum (FBS) (ExCell Bio, FSP500) for cell culture. In specific experiments, the cells were subjected to MG132 treatment (10 µM) (Selleck, Shanghai, China) and Cycloheximide (CHX, 710 µM).

### 4.3. Cell Transfections

The lentiviruses were obtained from Shanghai GeneChem Co., Ltd. (Shanghai, China). The GV492-GFP and GV115-EGFP lentiviral expression system (Shanghai GeneChem Co., Ltd.) was utilized for the transfection of cells expressing shRNA targeting RRS1 (sh-RRS1), control shRNA (sh-CON), scrambled control (OE-CON), and overexpressed RRS1 (OE-RRS1). The cells were subjected to infection with the corresponding lentiviruses at a multiplicity of infection (MOI) of 20 [37,38]. When the cells reached 20% to 30% confluence, they were transfected with the virus and Hitrans A&P (Shanghai GeneChem Co., Ltd.). Following 8 to 16 h of infection in a constant incubator, 2 mL of complete medium was replaced, and the cells were further cultured in a thermostatic incubator. Subsequent experimental procedures were conducted after 48 h of incubation. Small interfering RNA (siRNA) together with their scrambled sequences were synthesized by GenePharma Co., Ltd. The target sequence of GRP78 included forward 5' GAGGCUUAUUUGGGAAAGATT 3' and reverse 5' UCUUUCCCAAAUAAGCCUCTT 3'. Next, cells were subjected to transfection with siRNA through RNAFit (Hanbio Biotech Co., Ltd, Shanghai, China) for 48 h at the temperature of 37 °C, following the instructions provided by the manufacturer.

### 4.4. Quantitative Reverse Transcription Polymerase Chain Reaction (RT-qPCR)

The Trizol-based kit for RNA isolation (Vazyme, Nanjing, China) was used for total RNA extraction. The protocol provided by the manufacturer (Vazyme Corporation, Nanjing, China) was followed for RT-qPCR amplification. Below are the primer sequences used in this investigation: glyceraldehyde phosphate dehydrogenase (GAPDH) forward 5’-AGAAGGCTGGGGCTCATTTG-3’ as well as reverse 5’-AGGGGCCATCCACAGTCTTC-3’; GRP78 forward 5'-GACCCTTACTCGGGCCAAATT-3' as well as reverse 5’-GTAGAGCGGAACAGGTCCATGT-3'; RRS1 forward 5’-CCCTACCGGACACCAGAGTAA-3’ as well as reverse 5’ -CCGAAAAGGGGTTGAAACTTCC-3’.

### 4.5. Cell Proliferation Assay and Colony Formation Assay (CFA)

A density of 2 × 10^3^ cells/well was seeded into 96-well plates using BT549 and MDA-MB-231 cells. Following the initial incubation for 1, 2, 3, 4, and 5 days, each well was supplemented with a mixed solution of 10 µL cell counting kit 8 (Solarbio, Beijing, China) and 90 µL of medium. The mixture was carefully mixed, and subsequently incubated at an optimal temperature of 37 °C for an additional 2 h. In addition, the absorbance at the wavelength of 450 nm was detected through a microplate reader. For each treatment group, 500–1000 cells/well were plated in 6-well plates. Following two-week incubation at 37 °C and 5% CO2, colony formation was monitored daily, and the medium was regularly replaced. When the colonies reached a visible size, the culture media was carefully taken away. Subsequently, the cells underwent a fixation process involving 4% paraformaldehyde (PFA) for a duration of 20 min, followed by a thorough washing with phosphate-buffered saline (PBS). Subsequently, crystal violet (0.1%) staining was conducted for the fixed cells for half an hour. Finally, cells were washed and air-dried. Photographic documentation was conducted for further analysis.

### 4.6. Scratch Assay

The cells from each treatment group were distributed into 6-well plates with a cell density of 2 × 10^5^ cells/well. Upon reaching a confluence above 90% and forming a monolayer, a pipette gun head with a volume of 200 μL was adopted to produce a uniform cell-free wound area on the cell surface. After rinsing twice with PBS, 2 mL of serum-free DMEM medium was added for further culture. Photographs were taken at 0 h, 24 h, and 48 h to analyze cell migration systematically within the 48 h period.

### 4.7. Cell Invasion Assays

The inserts were treated with pre-coated Matrigel, which was diluted 40-fold (100 μL per well; Corning). Cells were cultured at a predetermined seeding density of 1 × 10^4^ cells/well using transwell inserts (Corning, Inc., New York, NY, USA.). The upper chamber was supplemented with serum-free medium as well as 1% penicillin/streptomycin, while the lower chamber contained 10% FBS and 1% penicillin/streptomycin. After 48 h of incubation, the transwell inserts were removed, and any non-invasive cells on top of the culture solution in the chambers were gently wiped away using a cotton swab. The inserts were then immersed in a 4% PFA solution for a duration of 20 min, stained with the crystal violet (0.1%) for half an hour, and photographed.

### 4.8. Co-Immunoprecipitation (Co-IP)

The protein A/G kit (Epizyme, Shanghai, China) was utilized for conducting Co-IP. The beads were activated after incubated in the lysis buffer (25 µL) overnight. After the appropriate treatment, the cells were subjected to homogenization in a meticulously prepared cell lysis buffer and kept at a consistent temperature of 4 °C for a duration of 30 min. The bicinchoninic acid (BCA) assay (Vazyme Corporation, Nanjing, China) was employed to quantify protein concentrations. Each lysate (approximately 500 µL) was subjected to an overnight incubation with either 4 µg of anti-GRP78 antibody (Proteintech, Cat No. 66574-1-Ig) or 1 µg of IgG (Bioss, Beijing, China, bs-0297R), along with the activated beads. Finally, the precipitate was treated with a lysis buffer. After the elution procedure, the IP was identified through Western blotting (WB) analysis.

### 4.9. Mass Spectrometry Analysis

Protein samples were digested in-gel at 37°C overnight with sequencing-grade modified trypsin (200ng; Promega, Madison, WI, USA). LC-MS/MS analysis of the digest was performed using an Obitrap-Elite mass spectrometer (Thermo Fisher Scientific, Waltham, MA, USA). Proteins were found by utilizing the Mascot search engine (version 2.3; Matrix Science, London, UK) and SEQUEST v.1.27 (University of Washington, Seattle, WA, USA) via the Proteome Discoverer software (version 1.4; Thermo Fisher Scientific, MA, USA) to look for the fragment spectra in the Swiss-Prot protein database.

### 4.10. Immunofluorescence (IF)

MDA-MB-231 and BT-549 cells were cultured at a density of 2000 cells/well on circular coverslips (Biosharp; cat. no. BS 14 RC). After treatment with a 4% PFA solution for 20 min, the mounted coverslips were washed three times with PBS. Following fixation, they were washed with phosphate-buffered saline solution (PBST) three times. A blocking treatment with 10% goat serum (Wuhan Boster Biological Technology, Ltd., Wuhan, China; cat. no. AR0009) was performed after cell infiltration with a 0.5% Triton X-100 solution for 10 min. Subsequently, coverslips were incubated overnight at the temperature of 4°C with anti-GRP78 (1:200, Proteintech; cat. no. 66574-1-Ig) and anti-RRS1 (1:200, Abcam; Catalog number ab188161) antibodies. The next day, cells were further treated with the appropriate fluorescent secondary antibodies (1:200, ABclonal; cat. no. AS039 and AS011) at room temperature for one hour, followed by three washes with PBST. Subsequently, 4',6-diamidino-2-phenylindole (DAPI) staining (Epizyme, Shanghai, China) was used to stain the nucleus, accompanied with an additional 10 min of incubation at ambient temperature, avoiding light. Finally, the cells were visualized and documented utilizing a laser confocal microscopy (Leica Stellaris 5; Leica Microsystems GmbH, Wetzlar, Germany).

### 4.11. Protein Stability Assay

After treating sh-CON alongside sh-RRS1 cells with 710 μM CHX at 80% confluence, protein samples were collected at predetermined intervals for WB analysis.

### 4.12. Western Blotting (WB) Analysis

The tissues that has been ground down or cells were treated with ristocetin-induced platelet aggregation buffer (Shandong Sparkjade Scientific Instruments Co., Ltd., Jinan, China) for total protein extraction. The BCA kit (Solarbio, Beijing, China) was then used for quantification. Subsequently, the protein samples (30–50 μg) were separated by 10% sodium dodecyl sulfate polyacrylamide gel electrophoresis (SDS-PAGE) and transferred onto polyvinylidene fluoride (PVDF)membranes (Millipore). After blocking the PVDF membranes with 5% skim milk for two hours, they were rinsed twice using TBST (tris buffered saline + 0.5% [v/v] Tween-20) buffer. Subsequently, the membranes were incubated overnight at 4 °C after the addition of primary antibodies, including RRS1 (1:2000; Abcam; cat. No. ab188161), GRP78 (1:5000; Proteintech; cat. No. ab32072), GAPDH (1:2000; Abclonal; cat. No. AC002), phosphorylated (p)-AKT (1:1000; Abmart; cat. No. T40067), p-PI3K (1:1000; Proteintech; cat. No. AP0427), PI3K (1:1000; Abclonal; cat. No. A4992), AKT (1:1000; Abmart; cat. No. T55561), and ubiquitin (1:1000; Proteintech; cat. No. 10201-2-AP). After incubation with specific secondary antibodies (1:5000; Abclonal; cat. No. AS014 and AS003) for one hour at room temperature, the visualization of PVDF membranes were conducted based on electrochemiluminescence (ECL) (Abclonal, Wuhan, China).

### 4.13. Statistical Analyses

For data analysis, SPSS v13.0 software (SPSS, Inc., CHI, USA) and GraphPad Prism8 (GraphPad Software, CAL, USA) were used. Data were described with mean ± standard deviation (SD). Student’s t-test was used for comparisons between two groups. On the other hand, one-way analysis of variance was utilized when comparing three or more groups. A p-value less than 0.05 was considered statistically significant.

## Data Availability

All data generated or analyzed during this study are included in this published article.
